# Exploring the mediating effect of feedback self-efficacy between students’ self-feedback behavior and academic proficiency

**DOI:** 10.3389/fpsyg.2025.1637028

**Published:** 2025-09-16

**Authors:** Yongle Yang, Zi Yan, Jinyu Zhu, Wuyuan Guo, Junsheng Wu, Bingjun Huang

**Affiliations:** ^1^Research Center for Basic Education Quality Development, School of Education, Jingchu University of Technology, Jingmen, China; ^2^Department of Curriculum and Instruction, The Education University of Hong Kong, Hong Kong, Hong Kong SAR, China; ^3^Shenzhen Baoan Haile Experimental School, Shenzhen, China

**Keywords:** self-feedback behavior, feedback self-efficacy, academic achievement, gender differences, multi-group SEM

## Abstract

Self-feedback and feedback self-efficacy are imperative components of self-regulated learning; few studies have investigated their combined impacts on academic achievement. This study examined the predictive effects of self-feedback behaviors, feedback self-efficacy, and academic proficiency using a questionnaire survey from 665 Chinese high school students across Chinese, English, and mathematics subjects. Structural equation modeling showed that only use feedback (UF) directly predicted academic proficiency, while both process feedback (PF) and use feedback (UF) demonstrated indirect effects mediated through feedback self-efficacy. At the same time, seek feedback (SF) was not a significant predictor in direct and indirect effect tests. Multi-group SEM analysis further explored gender differences in the effects; male students hold stronger predictive power of PF over feedback self-efficacy, while female students with feedback self-efficacy could achieve greater academic success. These results recognize the critical effects of feedback self-efficacy in translating students’ self-feedback behavior into their academic performance. The study empirically supports the self-system model and emphasizes the need for differentiated feedback instructional strategies among male and female students. It also contributes to scale studies of the recently published Self-feedback Behavior Scale (SfBS), by further supplementing evidence for its reliability and cross-gender applicability using a different dataset. The findings indicate that differentiated instructional strategies are necessary to empower students with more effective self-feedback strategies and personal beliefs; by doing this, students could better benefit from the feedback process and achieve substantial academic growth.

## 1 Introduction

Self-feedback has emerged as a salient construct in recent studies, reflecting students’ proactive engagement in seeking, processing, and using feedback to inform their academic decisions and actions (Nicol, 2021; Yang et al., 2025; Panadero and Lipnevich, 2022). Regardless of its theoretical prominence, empirical investigations into its effects on students’ academic proficiency remain fragmented, especially when considered alongside feedback self-efficacy (SFE), where students’ beliefs in their capacity to adopt feedback strategies in their learning process are considered. While feedback self-efficacy has demonstrated its effectiveness in the feedback reception process (Prilop et al., 2021; Johannes and Haase, 2024; Wang and Wu, 2008), few studies have explicitly examined how self-feedback behaviors and feedback beliefs interact to influence composite academic performance across multiple disciplines, such as Chinese language, English, and mathematics. This gap is particularly pertinent in high-stakes East Asian contexts, where sociocultural norms and exam pressures often shape the interplay between feedback engagement and academic success (Brown et al., 2012; Lipnevich and Smith, 2022; Vattøy, 2020).

Moreover, gender has also been identified as a potential moderator in feedback learning processes. Studies indicate that female students may exhibit higher feedback engagement and self-efficacy levels, particularly in language domains, though these effects vary across subjects and cultural contexts (Adams et al., 2020; Ahn et al., 2016; Huang, 2013). Understanding the gender differences can inform differentiated instructional practices and feedback strategies tailored to learners’ motivational profiles (Narciss et al., 2014; Virtanen and Nevgi, 2010).

To attempt the research gaps, this study investigates the effects of self-feedback behavior, feedback self-efficacy, and their academic performance within a representative sample of Chinese high school students. Specifically, this study examines whether feedback self-efficacy mediates the effect between three dimensions of self-feedback—seeking, processing, and using feedback, and academic achievement across core subjects. Furthermore, it also investigates the predictive effects of gender on self-feedback behavior and academic proficiency through the mediating role of feedback self-efficacy. This study aims to deepen our knowledge about how students’ self-feedback behaviors and their feedback beliefs shape their academic success, thereby offering insights and implications for enhancing feedback learning strategies in classroom instruction in the context of Chinese high schools.

## 2 Literature review

### 2.1 Self-feedback behavior

The growing emphasis on student-centered feedback has drawn attention to the vital role of students’ proactive engagement in the self-feedback process (Winstone et al., 2017; Malecka et al., 2022; Winstone et al., 2019; Panadero and Lipnevich, 2022). Scholars have increasingly attempted to investigate the effects of self-feedback mechanisms, with two noteworthy frameworks offering considerable insights. One framework is Nicol’s (2021) conceptualization of “internal feedback,” which underscores the value of comparing teacher comments with learners’ prior experiences and academic goals. Another significant contribution stems from Panadero et al. (2024), who have conducted multiple empirical investigations and proposed a multi-phase self-feedback model derived from over 500 self-assessment observations across various disciplines and education levels, describing the self-feedback shall encompass six cognitive processes, in this conceptual model, students start with engaging with external comments through monitoring and inquiring, followed by evaluative comparison with exemplar work, and ideally proceed to revise their own learning assignments. This model offers a clear behavioral roadmap for self-feedback. However, it was created in controlled research environments rather than in authentic classroom contexts, and it focuses on the internal feedback generation process rather than its future use for learning improvement purposes (Panadero et al., 2024; Yang et al., 2025).

More recently, scholars have framed the self-feedback behavioral model from a learner-centered perspective, emphasizing students’ active role in seeking, processing, and using feedback for academic growth (Malecka et al., 2022; Yan and Carless, 2022). A cyclical behavioral model has been proposed, outlining how students intentionally engage in a self-feedback process, it views self-feedback not as a single action but as an ongoing cyclical process of refinement, where students continually interact with external comments, evaluate their feedback quality, and formulate their future learning improve plans accordingly (Carless and Boud, 2018; Panadero et al., 2019; Yang et al., 2025). Proactive self-feedback engagement appears to improve students’ feedback self-efficacy and learning performance (Panadero et al., 2017; Panadero et al., 2024; van der Kleij, 2020).

### 2.2 Feedback self-efficacy

Feedback self-efficacy is conceptualized as students’ belief in their competence to meaningfully interpret, evaluate, and implement feedback to achieve academic success (Adams et al., 2020; Brown et al., 2012). It highlights the dynamic connections between personal motivation, behavioral engagement, and environmental impacts in their learning process (Bandura, 2001; Schunk, 1989; Karl et al., 1993; Wang and Wu, 2008). In the context of classroom instruction, this extends academic self-efficacy into a more specific domain of feedback studies, where feedback is seen as a critical component in students’ academic success (Karl et al., 1993; Nease et al., 1999; Smit et al., 2023).

In exam-oriented learning environments such as Chinese high schools, feedback plays a central role in shaping students’ learning behaviors in different subject domains (Gong et al., 2025; Gan et al., 2021; Yang and Yang, 2018). Whether processing corrective comments in mathematics problem-solving, revision of English and Chinese writing assignments, students should purposely and proactively devote themselves to the learning process with multi-sourced feedback; they also need to have the personal belief that they can translate the feedback into improved learning outcomes. Research suggests that students with higher feedback self-efficacy are more likely to engage in the active feedback process across subjects (Adams et al., 2020; Brown et al., 2016; Chan and Lam, 2010).

Moreover, enhancing students’ feedback self-efficacy has improved academic outcomes, particularly when integrated with instructional strategies that promote mastery experiences, peer modeling, and structured reflection (Brown et al., 2016; Chan and Lam, 2010). This study conceptualizes feedback self-efficacy as a key mediating role in determining whether students’ feedback engagement in seeking, processing, or using feedback is successfully translated into composite academic success across Chinese, English, and mathematics domains.

### 2.3 Effects between self-feedback, feedback self-efficacy, and academic achievement

While numerous studies have investigated the effects of self-feedback, feedback self-efficacy, and academic achievement, few studies have integrated these constructs within a uniform theoretical framework, particularly in multi-subject secondary education contexts such as Chinese high schools (Hao and Razali, 2022; Wardana et al., 2025; Zhan et al., 2023; Zhu et al., 2024). This gap is significant given that self-feedback behaviors and feedback beliefs are increasingly recognized as essential components of self-regulated learning, which directly shape students’ academic trajectories across diverse domains, including language and mathematics (Bernacki et al., 2015; Vattøy, 2020; Yang et al., 2025).

Students’ active engagement in the self-feedback process could enhance their feedback beliefs, wherein they monitor, evaluate, and adjust their learning strategies based on performance comments they received and processed (Winstone et al., 2017; Zou et al., 2023; Panadero et al., 2019). However, the extent to which the self-feedback behavior contributes to academic success is pertinent to students’ confidence in their ability to process and use feedback effectively. In this process, feedback self-efficacy is a motivational mediator that translates self-feedback actions into performance outcomes by fostering perseverance, strategic planning, and task-specific effort (Lee and Evans, 2019; Raaijmakers et al., 2019). Research works have also examined that academic self-efficacy is a significant predictor of learning motivation and achievement across subject areas (Dogan, 2015; Lee et al., 2014).

Despite these insights, few studies have empirically investigated the mediating role of feedback self-efficacy in the effect between self-feedback and composite academic performance across subjects such as Chinese language, English language, and mathematics. This study attempts to close this gap by employing the self-system model, which argues that people’s internal beliefs (e.g., self-efficacy) mediate behavioral engagements (e.g., feedback) on academic outcomes (Connell and Wellborn, 1991; Yang et al., 2023). By examining these predictive effects, this study attempts to reveal the cognitive and motivational mechanisms through which self-feedback engagement contributes to academic success in high-pressure educational settings.

### 2.4 Gender difference in self-feedback and feedback self-efficacy

Gender has emerged as a critical moderator in shaping how students interact with self-feedback behaviors and feedback self-efficacy processes within learning contexts (Panadero et al., 2020; Zhang H., 2024; Zhang Y., 2024). Empirical studies suggest that female students engage more actively into the feedback process, often showing more intention in seeking, and using feedback sourced from their teachers and peers (Miller and Karakowsky, 2005; Guo and Zhou, 2021), while male students are more inclined to emphasize on these performance-oriented feedback, and are more likely to benefit from the reflective feedback process (Johnson et al., 1993; Wang et al., 2023) Additionally, existing research reports inconsistent evidence regarding gender effects in how students perceived their capacities in the feedback learning processes (Guo, 2021; Yu and Deng, 2022). While some studies report that female students often hold stronger beliefs in their self-feedback capacities (Huang, 2013; Panadero et al., 2020), others argue that there are non-significant gender differences in students’ confidence in their capacities in using feedback in their learning process, particularly within EFL learning environments (Hoogerheide et al., 2016; Guo, 2021; Yu and Deng, 2022). These inconsistencies imply that the gender effects among students’ self-feedback behaviors and feedback self-efficacy deserve more empirical exploration. Through more tailored feedback interventional strategies in alignment with students’ gender differences, teachers can create more inclusive and equitable feedback learning environments that promote more effective self-feedback behavioral engagement and leverage the benefits of self-feedback in their learning process more effectively.

### 2.5 The present study

Given the literature review of the effects of students’ self-feedback behavior, feedback self-efficacy, and their academic performance, three research questions (RQs) would be examined in this study.

*RQ1*: What are the effects of students’ self-feedback behavior on academic performance?

*Hypothesis 1*: Seeking feedback (H1.1), processing feedback (H1.2), and using feedback (H1.3) will positively predict students’ academic performance.

*RQ2*: What is the effect of students’ feedback self-efficacy on academic performance?

*Hypothesis 2*: Feedback self-efficacy (H2) will positively predict students’ academic performance.

*RQ3*: To what extent does feedback self-efficacy mediate the effect between self-feedback behavior and academic performance?

*Hypothesis 3*: Seeking feedback (H3.1), processing feedback (H3.2), and using feedback (H3.3) will predict students’ feedback self-efficacy.

*Hypothesis 4*: Feedback self-efficacy will mediate the effects of seeking feedback (H4.1), processing feedback (H4.2), and using feedback (H4.3) on their academic performance.

The present study also supplemented additional evidence for the scale development and validation of the recently validated questionnaire of the Self-feedback Behavior Scale (SfBS; Yang et al., 2025) using an independent dataset. This study would further support the validity and reliability of the SfBS.

## 3 Methodology

To investigate the hypothesized effects, a convenience sample using a cross-sectional dataset was collected to explore the predictive effects between students’ self-feedback behavior, feedback self-efficacy, and their academic performance. The quantitative dataset was collected through a self-reported questionnaire and analyzed through structural equations modeling. This study intends to extrapolate findings from a representative sample of Chinese students to the broader context, enabling valid inferences about their perceptions of self-feedback behaviors, perceived feedback self-efficacy, and academic proficiency.

### 3.1 Participants

A random sampling method was used to recruit participants from four high schools in Shenzhen, comprising two public and two private schools. A total of 698 students aged 15–18 years completed the questionnaire. This study focused on high school students since, first, existing feedback studies have predominantly examined tertiary education settings (Malecka et al., 2022; Molloy et al., 2020b), while the feedback studies in secondary educational settings were relatively under explored; second, students at primary and lower secondary levels may have difficulty comprehending the constructs of self-feedback behavior and feedback self-efficacy, which could compromise the accuracy of their responses. Additionally, the relatively small proportion of Grade 12 participants might be attributed to the intensive learning pressure associated with the Gaokao—the national university entrance examination administered at the end of Grade 12.

In structural equation modeling (SEM), the sample size could be determined using a rule of thumb with the ratio of cases to free parameters (N:q). In contrast, a minimum ratio of 10:1 is often acceptable (Kyriazos, 2018). However, Kline (2023) advocates for a more stringent ratio of 20:1. Therefore, given that the present instrument comprised 15 items, a minimum of 300 participants was needed for SEM analysis. Moreover, as this study conducted multi-group SEM to examine predictive effects in different gender groups, a minimum of 300 valid responses per group was believed to ensure adequate statistical power. Additionally, given the nature of potential gender imbalances in targeted participants and unforeseeable student absences during the data collection period. On the one hand, it was argued that the large sample (*N* > 500) might inflate the chi-square statistics (Barrett, 2007). We then mitigated this concern using multiple fit indices (CFI, TLI, RMSEA, and SRMR) in the model fit evaluation process. On the other hand, a large sample (*N* > 500) could provide more stable estimates, enhance generalizability, and strengthen the validity of invariance testing (Asparouhov and Muthén, 2009). Eventually, 698 students were surveyed in this study (see Table 1).

**Table 1:** The demographic information of participants.

	Frequency	Percentage (%)
Year level		
G10	294	44.21%
G11	262	39.40%
G12	109	16.39%
Gender		
Male	347	52.18%
Female	318	47.82%
*N*	665	100.00%

### 3.2 Measures

#### 3.2.1 Self-feedback Behavior Scale

Self-feedback behavior was measured using the Self-feedback Behavior Scale (SfBS) developed by Yang et al. (2025), comprising 11 items that capture three theoretically grounded components of self-feedback behavior: seeking feedback (SF; 4 items, e.g., “I seek out examples of high-quality work to enhance my own work”), processing feedback (PF; 3 items, e.g., “I carefully evaluate the feedback I receive before deciding whether to incorporate it”), moreover, using feedback (UF; 3 items, e.g., “I can create a learning improvement plan based on clear inferences from feedback”). These components reflect the tripartite framework of self-feedback behavioral engagement—seeking, processing, and using feedback—as conceptualized in the self-feedback behavioral model (Yang et al., 2025).

#### 3.2.2 Feedback self-efficacy

The feedback self-efficacy (FSE) construct in the context of self-feedback was operationalized through four items adapted from the Chinese version of the General Self-efficacy Scale (Zeng et al., 2022), initially developed by Schwarzer and Jerusalem (1995). These adapted items were specifically contextualized to reflect students’ perceived competence in executing self-feedback behaviors. They included “I know how to implement self-feedback” and “I know how to approach others for their comments.”

#### 3.2.3 Academic performance

Academic success is imperative in assessing students’ learning growth; it could also be a critical indicator for their future academic opportunities (Cheng and Hamid, 2025). This study adopts the composite Chinese Language, English, and Mathematics scores to represent their academic performance since these three subjects are widely recognized as the foundational components of China’s national curriculum (Zhang H., 2024; Zhang Y., 2024). Moreover, this combination of subjects could also represent the cognitive development goals and the priorities of public educational services in the educational settings (Zou and Zhu, 2023; Wu et al., 2025). Specifically, the Chinese Language subject is instructed to develop students’ language literacy, reading comprehension, and contextual reasoning skills (Zhao et al., 2021). Conversely, mathematics aims to enhance students’ logical thinking, problem-solving ability, and quantitative reasoning competencies (Zhang et al., 2021). The English Language subject is taught to improve students’ English proficiency and cross-cultural communicational capacities (Xie, 2024; Zhang H., 2024; Zhang Y., 2024). Furthermore, these three subjects are taught as essential subjects and counted as mandatory components in the Gaokao, thus substantially influencing their future college admission results (Cheng and Curtis, 2010; Cao and Chen, 2025). Additionally, these three subjects are instructed and examined periodically at a national scale (Wang et al., 2024); therefore, they could enhance the generality of the findings and implications of this study.

### 3.3 Procedure

The questionnaire was evaluated by psychological assessment professionals as well as frontline teachers; it was aimed to ensure the questionnaire content was unambiguously and unbiasedly presented. Furthermore, ethical approval was obtained from the Human Research Ethics Committee (HREC) before the questionnaire packages were distributed to target participants. A comprehensive questionnaire package—comprising (a) informed consent forms, (b) demographic information, (c) the Self-feedback Behavior Scale, (d) the Feedback Self-efficacy Scale, and (e) self-reported cumulative academic scores—was distributed to target students.

All scales were administered in simplified Chinese to ensure linguistic and cultural appropriateness. The scales adopted a six-point positively described Likert format (1 indicates strongly disagree, while six indicates strongly agree), as Lam and Klockars (1982) suggested. This format was chosen to accommodate Chinese students’ cultural predisposition toward affirmative responses and to enhance variability in response patterns (Brown and Harris, 2013).

### 3.4 Data analysis

The collected dataset was screened before formal statistical analysis. Consistent with the recommendations of Tabachnick and Fidell (2007), no entries were removed as item-level missing data remained below 5%. Moreover, applying the Mahalanobis distance rule with a significance threshold of 0.95 identified 33 multivariate outliers, which were subsequently removed. Therefore, the obtained valid sample size is 665 for future analysis. Furthermore, the normality of the dataset was assessed with skewness ranging from −0.42 to −0.14 (within the ±3 criteria) and kurtosis between −0.04 and 0.47 (within the ±10 criteria), indicating the final dataset was suitable for structural equation modeling (SEM) studies (Kline, 2023).

Subsequently, the internal consistency was assessed using McDonald ω, with values of 0.70 or higher considered acceptable (Hayes and Coutts, 2020; McDonald, 2011). Confirmatory factor analysis (CFA) with classical maximum likelihood estimation (MLE) was conducted to evaluate the adequacy of the measurement models. The following criteria were considered when evaluating the model fit: TLI and CFI > 0.90, RMSEA < 0.06, and SRMR < 0.08 (McNeish and Wolf, 2023; Schreiber et al., 2006). The mediating effect analysis examined the indirect effects through feedback self-efficacy using bootstrapping with 5,000 samples and a 95% confidence interval. Eventually, multivariate SEM was used to examine the hypothesized effects between self-feedback behaviors, feedback self-efficacy, and academic proficiency. Given the previous arguments that gender affects feedback engagement and academic outcomes (Irvine, 1986; Yan, 2016; Guo, 2020; Panadero et al., 2020), multigroup SEM analysis was conducted to further investigate and explore the gender effects in the hypothesized effects. All preliminary data screening, validity, and reliability tests were accomplished using SPSS 26.0, while CFA and SEM studies were conducted using the lavaan package in R (R Core Team, 2019).

## 4 Results

The findings are presented following the data analysis procedure. First, the means, standard deviations, and correlations between each pair of dimensions were reported; this provided evidence of the central tendencies and interconnections between constructs. Meanwhile, the validity and internal consistency of each construct were also examined. Second, CFA technique was employed to evaluate the measurement model fit for three hypothesized models: Model 1, which examined the direct predictive effect between self-feedback behaviors and academic performance; Model 2, where feedback self-efficacy was treated as a mediating factor between self-feedback behaviors and academic achievement; and Model 3, multi-group CFA was used to investigate the hypothesized mediating effects of feedback self-efficacy across different gender groups. Third, SEM analysis was employed to investigate the hypothesized structural predictive effects in different conceptual models, specifically examining the direct and indirect effects of self-feedback behaviors on academic outcomes, and whether these pathways differentiated due to gender differences. This data analysis report was also consistent with the research questions and hypotheses in the present study.

### 4.1 Preliminary analysis

Table 2 presents the descriptive statistics and bivariate correlations for the study factors. Among the three dimensions of self-feedback behavior, participants reported the lowest level of agreement with use feedback (UF) (M = 4.19), and the highest with process feedback (PF) (M = 4.54), indicating differential engagement across self-feedback stages. All variables demonstrated statistically significant and positive intercorrelations, providing preliminary empirical support for the theoretical framework employed to investigate the effect of students’ self-feedback processes on academic outcomes.

**Table 2:** Descriptive results and correlations between factors.

Factor	FSE	SF	PF	UF
FSE	—			
SF	0.45^***^	—		
PF	0.48^***^	0.68^***^	—	
UF	0.46^***^	0.68^***^	0.62^***^	—
Mean	4.42	4.27	4.54	4.19
SD	0.97	0.97	0.95	1.01
Skewness	−0.42	−0.15	−0.42	−0.14
Kurtosis	0.47	−0.04	0.32	−0.16

FSE, feedback self-efficacy; SF, seek feedback; PF, process feedback; UF, use feedback. ^*^p < 0.05, ^**^p < 0.01, and ^***^p < 0.001.

The internal consistency was also assessed using McDonald’s omega coefficients across each dimension to assess the psychometric properties of the measurement model. The values ranged from 0.802 for use feedback (UF) to 0.928 for feedback self-efficacy (FSE), with an overall reliability coefficient of 0.93, reflecting strong internal reliability across the measurement instruments (see Table 3).

**Table 3:** Item number and McDonald ω for each resultant scale.

Scale	Number of items	McDonald ω
FSE	4	0.928
SF	4	0.861
PF	3	0.896
UF	4	0.802
Subtotal	15	0.951

FSE, feedback self-efficacy; SF, seek feedback; PF, process feedback; UF, use feedback.

### 4.2 Confirmatory factor analysis

The model fit of the three proposed models, based on 15 items, was evaluated and is summarized in Table 4. Across all models, the fit indices demonstrated adequate levels, though the *χ*^2^/df ratio of Model 1 appeared above the benchmark, likely due to the comparatively large sample size (Hu and Bentler, 1999).

**Table 4:** Report of fit indices for three models.

Model	*χ* ^2^	df	*χ*^2^/df	*p*	CFI	TLI	RMSEA	SRMR	Difference test		
Criteria	/	/	<3	<0.001	>0.90	>0.90	<0.06	<0.08	Δ*χ*^2^	Δdf	*p*
Model 1	147.23	39	3.775	<0.001	0.974	0.964	0.065	0.029			
Model 2	227.06	81	2.803	<0.001	0.979	0.973	0.052	0.027			
Model 3	256.669	95	2.702	<0.001	0.977	0.971	0.051	0.033	29.609	14	0.009

Furthermore, all standardized factor loadings for the latent variables were statistically significant (*p* < 0.001), affirming the validity of the factorial structure for each model. Collectively, these results provide strong empirical support for the construct validity of the measurement models.

### 4.3 Structural equations modeling analysis

Following the validation of measurement models, structural equation modeling (SEM) was employed to test the hypothesized structural effects across the three conceptual models. Specifically, the analysis examined the direct and indirect effects of students’ feedback self-feedback behaviors on their academic proficiency. Standardized regression coefficients for each model are presented individually, facilitating comparative evaluation of the hypothesized direct and mediated pathways.

#### 4.3.1 Report on the predictive effect between feedback, self-feedback, and performance (RQ1)

**Figure 1:**
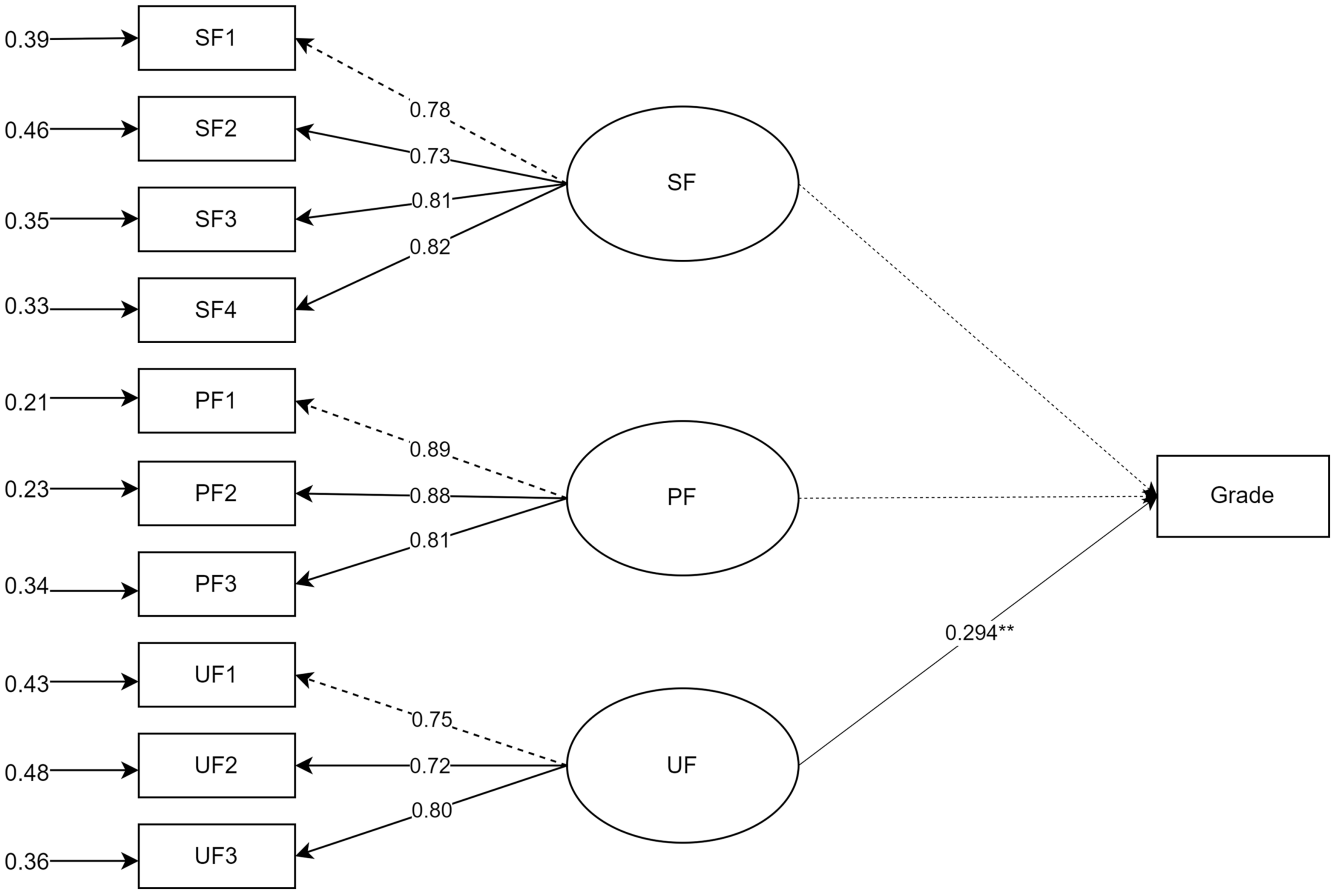
SEM analysis of Model 1.

In Model 1, neither seek feedback (SF) nor process feedback (PF) emerged as significant predictors of academic proficiency. However, use feedback (UF) demonstrated a statistically significant and moderate predictive effect (*β* = 0.275), indicating that only Hypothesis H1.3 was empirically supported, while H1.1 and H1.2 were not. This model accounted for 18.1% of the variance in students’ academic performance (see Figure 1).

#### 4.3.2 Report on the mediating role of feedback self-efficacy between self-feedback and performance (RQ2 and RQ3)

**Figure 2:**
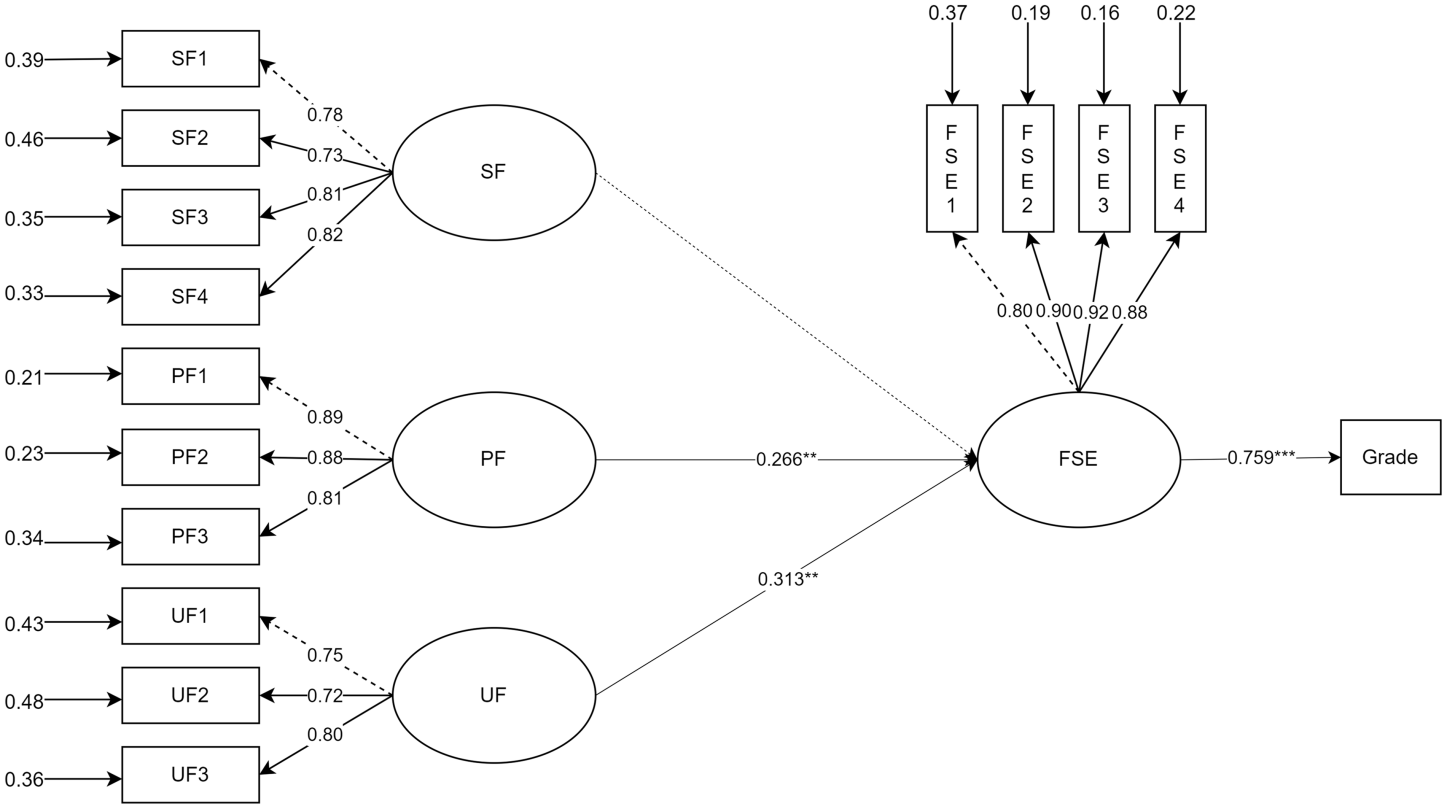
SEM analysis of Model 2.

In Model 2, both PF and UF demonstrated significant predictive power on students’ feedback self-efficacy, whereas SF did not demonstrate a statistically significant predictor. Feedback self-efficacy, in turn, showed a substantial predictive effect on students’ academic proficiency. These findings lend empirical support to Hypotheses H2, H3.2, and H3.3, while H3.1 was not supported. Furthermore, UF demonstrated a stronger predictive power on self-efficacy than PF, suggesting variation in the relative strength of feedback actions. The structural model explained 32.3% of the variance in feedback self-efficacy and 57.7% in academic proficiency (see Figure 2).

#### 4.3.3 Report the indirect effects on academic proficiency

As shown in Table 5, indirect effect analyses further revealed that both PF and UF exerted statistically significant indirect impacts on academic performance mediated by feedback self-efficacy. In contrast, SF did not show a significant mediating pathway. These results could support the Hypotheses H4.2 and H4.3, whereas H4.1 was not supported. Regarding effect size, the indirect influence of UF (*β* = 0.237) was marginally greater than that of PF (*β* = 0.202), underscoring UF’s relatively stronger mediating role through feedback self-efficacy.

**Table 5:** Report of hypotheses of direct and indirect effects on academic proficiency.

Hypothesis	Unstd. *b*	St. *β*	Std. error	95% CI	
				LL	UL
Direct effects					
H1.1: SF → Grade	2.076	0.097	0.104	−0.108	0.301
H1.2: PF → Grade	1.357	0.066	0.080	−0.090	0.222
H1.3: UF → Grade	6.269^**^	0.294^**^	0.100	0.097	0.490
H2: FSE → Grade	0.955^***^	0.759^***^	0.028	0.705	0.814
H3.1: SF → FSE	0.034	0.033	0.092	−0.147	0.213
H3.2: PF → FSE	0.238^**^	0.266^**^	0.070	0.129	0.402
H3.3: UF → FSE	0.330^**^	0.313^**^	0.083	0.150	0.475
Indirect effects					
H4.1: SF → FSE → Grade	0.032	0.025	0.070	−0.111	0.162
H4.2: PF → FSE → Grade	0.227^***^	0.202^***^	0.054	0.095	0.308
H4.3: UF → FSE → Grade	0.315^***^	0.237^***^	0.064	0.113	0.362
Total effects					
SF → Grade	0.128	0.100	0.095	−0.086	0.287
PF → Grade	0.089	0.079	0.073	−0.064	0.223
UF → Grade	0.366^**^	0.276^**^	0.086	0.107	0.445

FSE, feedback self-efficacy; SF, seek feedback; PF, process feedback; UF, use feedback; CI, confidence level; LL, lower level; UL, upper level. ^*^p < 0.05, ^**^p < 0.01, and ^***^p < 0.001.

#### 4.3.4 Report on the gender difference in the mediating effects of feedback self-efficacy between self-feedback behavior and academic performance

Furthermore, multi-group structural equation modeling (MG-SEM) was conducted to examine whether the hypothesized mediation pathways differed by gender. Prior to the multi-group SEM analysis, a multi-group CFA was conducted to test measurement invariance across gender. When factor loadings were constrained to be equal, the chi-square (Δ*χ*^2^) change was non-significant; however, given the sensitivity of chi-square to large sample size in this study, additional fit indices were examined. The changes in CFI (ΔCFI = 0.008) and SRMR (ΔSRMR = 0.015) both met recommended thresholds (ΔCFI ≤ 0.01; ΔSRMR ≤ 0.03) for establishing metric invariance (Cheung and Rensvold, 2002; Chen, 2007). Following Byrne’s (2012) guidelines, these results indicated that metric invariance was achieved, supporting further multi-group SEM analysis. The model demonstrated a satisfactory fit to the data, with a chi-square value of *χ*^2^ = 256.67, degrees of freedom (df) = 95, and a relative chi-square of *χ*^2^/df = 2.702 (*p* < 0.001). Additional fit indices met recommended thresholds: CFI = 0.977, TLI = 0.971, RMSEA = 0.051, and SRMR = 0.033. A chi-square difference test comparing Model 3 (with gender difference) with Model 2 (without gender difference) yielded a statistically significant improvement (Δ*χ*^2^ = 29.609, Δdf = 14, *p* = 0.009). This result indicates that incorporating gender as a moderating variable substantially enhanced the model’s explanatory power.

Consistent with the findings from Model 2, both male and female sub-groups exhibited parallel predictive structures. Specifically, process feedback (PF) and use feedback (UF) significantly predicted feedback self-efficacy in both groups, and feedback self-efficacy significantly predicted academic proficiency across genders. At the same time, seek feedback (SF) could not produce statistically significant predictive power on feedback self-efficacy in both groups. These results support the generalizability of the core mediation model across male and female students.

However, differences emerged in the predictive strength of these effects. The predictive power of PF to feedback self-efficacy among male students was comparatively more substantial. In contrast, the predictive power of UF on FSE and FSE on academic proficiency was more pronounced among female students (see Table 6).

**Table 6:** Report of direct effects across gender groups.

Group	Direct effects	Unstd. *b*	St. *β*	Std. error	95% CI	
					LL	UL
M	FSE → Grade	16.523^***^	0.729^***^	0.032	0.667	0.792
	SF → FSE	0.032	0.034	0.100	−0.161	0.229
	PF → FSE	0.252^***^	0.278^***^	0.074	0.132	0.424
	UF → FSE	0.296^***^	0.308^***^	0.089	0.133	0.483
F	FSE → Grade	16.523^***^	0.776^***^	0.030	0.718	0.834
	SF → FSE	0.032	0.031	0.092	−0.149	0.211
	PF → FSE	0.252^***^	0.259^***^	0.068	0.125	0.393
	UF → FSE	0.296^***^	0.306^***^	0.083	0.144	0.467

FSE, feedback self-efficacy; SF, seek feedback; PF, process feedback; UF, use feedback; CI, confidence level; LL, lower level; UL, upper level. ^*^p < 0.05, ^**^p < 0.01, and ^***^p < 0.001.

## 5 Discussion

The present study aimed to investigate the structural effects of self-feedback behavior, feedback self-efficacy, and academic proficiency within the context of Chinese high school students. The initial analysis focused on examining the direct effects of each dimension of self-feedback behavior on students’ academic achievement (Model 1), drawing upon previous studies that posit self-feedback as influential in enhancing academic outcomes (Butler and Winne, 1995; Brown et al., 2016). Subsequently, the study examined whether feedback self-efficacy functioned as a mediating role in the effect between self-feedback behaviors and academic performance, thereby supplementing a more nuanced understanding of the educational processes underlying effective learning behaviors.

Furthermore, to explore the potential impact of gender differences, the structural effects were further examined through multi-group comparisons based on different gender groups, assessing whether the predictive pathways differed between male and female students. This section revisits the primary research questions of the present study. It comprehensively explains the empirical findings, situating them within the broader context of existing theoretical frameworks and empirical studies.

### 5.1 Self-feedback behavior and academic performance

Regarding the first hypotheses (H1.1 to H1.3), we assumed that each action of self-feedback, namely, seek feedback (SF), process feedback (PF), and use feedback (UF), would serve as a significant predictor of students’ academic proficiency. At the same time, numerous studies argued that not all feedback-related behaviors could necessarily produce academic attainments (Butler and Winne, 1995; Hattie and Timperley, 2007; Nicol and Macfarlane-Dick, 2006). Our findings showed that, within three self-feedback actions, only UF could significantly determine academic proficiency, whereas neither SF nor PF could predict students’ academic outcomes significantly. These findings implied that while self-feedback behavioral engagement is often treated as an effective learning strategy, only students who purposefully and tactically use it in their future learning improvement scheme, rather than merely obtaining or processing it, could contribute to learning enhancement. This finding was consistent with recent feedback arguments, which differentiate feedback reception from acting upon feedback (Carless and Boud, 2018; Winstone and Carless, 2019). Research has shown that the benefits of feedback are often contingent on students’ willingness and capacity to translate received feedback into feasible learning actions (Cohen and Singh, 2020; Briscoe et al., 2023). In this context, UF behaviors reflect a learner’s ability to make concrete adjustments, set new goals, and re-formulate learning strategies based on processed feedback—behaviors more closely aligned with outcome-based performance.

One possible explanation for the limited impact of SF and PF is that students may seek or cognitively engage with feedback without necessarily internalizing or acting upon it. In highly exam-oriented learning environments like Chinese high schools, students often prioritize learning outcomes over the process, which may limit the depth of reflective feedback processing or strategic seeking behaviors (Gong et al., 2025; Kirkpatrick and Zang, 2011). Another possible reason is that students might not effectively understand the meaning of feedback-seeking in their learning experiences, given its complex nature, which could constrain their interpretations and judgment of how feedback-seeking behaviors could impact their learning outcomes. Moreover, feedback-seeking may occur passively (e.g., teacher-driven) rather than reflect genuine self-regulated effort, reducing its effectiveness as a predictor of achievement.

These findings suggest that promoting feedback-seeking or processing behaviors in isolation may be insufficient. Instead, educational interventions should focus on cultivating students’ capacity to implement feedback meaningfully, through goal setting, revision, and performance monitoring. Teachers and curriculum experts should consider how to scaffold students’ feedback literacy, not just in understanding feedback, but in applying it effectively to optimize academic achievement.

### 5.2 Feedback self-efficacy and academic performance

The second hypothesis (H2), where students’ feedback self-efficacy was assumed to predict academic proficiency significantly, was supported by our findings. Specifically, students who were more confident in their competence to interpret and use feedback were likely more successful in learning attainment. This finding supports the previous argument that personal feedback beliefs are critical in determining individuals’ motivation, behaviors, and performance (Bandura, 1997). In the context of classroom instructions, feedback self-efficacy emerges as an imperative role, enabling students to transform self-feedback actions into enhanced learning outcomes. Feedback self-efficacy describes students’ belief in their capacity to effectively engage with feedback in their learning process. It seems it could enable students to translate their self-feedback behavioral engagement into learning outcomes effectively. Similarly, Panadero et al. (2024) found that students’ self-feedback behaviors could significantly improve their confidence in employing feedback in their future learning.

In Chinese high schools, where students are under intensive learning pressure, feedback self-efficacy might supplement their psychological support in their behavioral engagement in the self-feedback process. Students with higher feedback self-efficacy are more likely to obtain comments from their teachers and peers proactively, make evaluative judgements about the comments they received, and use these comments to reflect their learning performance as well as to re-formulate their future learning schemes, by doing this, their learning performance could be enhanced (Panadero et al., 2024; Yang et al., 2025; Yan and Brown, 2017). This echoes previous arguments that feedback might be insufficient in improving students’ learning outcomes unless it could improve their confidence and competence to engage with it proactively (Winstone et al., 2017; Carless and Winstone, 2020). In short, this finding supports the imperative role of feedback self-efficacy as a cognitive enabler of academic success (Doménech-Betoret et al., 2017; Zheng et al., 2021). Henceforth, future feedback interventions aimed to enhance students’ learning performance should also consider the instructional strategies to improve students’ confidence in their self-feedback knowledge and skills (Tai et al., 2018; Molloy et al., 2020a; McGinness et al., 2020).

### 5.3 Mediating effects of feedback self-efficacy

The present study supplements empirical evidence to support the mediating role of feedback self-efficacy in navigating students’ self-feedback behaviors and their academic success. Notably, both PF and UF showed significant predictive effects on academic proficiency through feedback self-efficacy, whereas SF could not impact academic proficiency through feedback self-efficacy. These findings echo previous arguments that the mechanism of feedback in determining academic outcomes is a complex cognitive process, where students’ personal beliefs about their capacities to adopt feedback strategies effectively are also critical (Goetz et al., 2016; Hinduja et al., 2024; Keller et al., 2024).

This mediating effect aligns with the social cognitive framework of self-regulated learning, which posits that cognitive, motivational, and behavioral components interact to influence academic performance (Zimmerman, 2000; Butler and Winne, 1995). Within this framework, feedback self-efficacy is a pivotal motivational mechanism; students strongly believe they can engage in self-feedback when they constantly process and use feedback strategies in their learning. Additionally, their learning outcome could be significantly improved with a more substantial perception of feedback beliefs.

Furthermore, the differentiated impact of self-feedback actions on academic proficiency through feedback self-efficacy also indicates that self-feedback is a complicated cognitive process where each action contributes different values. Specifically, PF and UF contribute significantly to enhancing feedback self-efficacy, while SF is insufficient in determining students’ beliefs in their feedback capacities. Therefore, future feedback interventional programs should emphasize the strategies to improve students’ knowledge and capacities to enhance their processing and use of feedback practices (Winstone et al., 2021; Yang and Zhang, 2023).

Considering the magnitude of the indirect effect of UF over PF on academic performance, it seems that students are more likely to emphasize the critical role of using a feedback strategy in their learning process compared to the effects of processing feedback. This is consistent with the feedback engagement model proposed by Carless and Winstone (2020), which argues that students should appreciate and implement feedback to maximize its benefits. Once the obtained feedback could be translated into concrete learning improvement strategies effectively, for example, correct their mistaken assignment in the short term or re-formulate their future learning goals and initiate learning improvement plans, goal setting or mistaken assignment correction, students are likely to benefit from this process with more significant academic growth (Brown et al., 2016; Yang and Zhang, 2023).

In summary, the present study empirically supports the critical role of feedback self-efficacy in mediating the effects of students’ self-feedback behaviors and academic achievement. Conventional feedback instructional interventions, which motivate students’ behavioral engagement, might be insufficient unless their personal beliefs about self-feedback can also be improved. Additionally, instructional designs that could facilitate students’ reflective evaluation of feedback and further take action upon it are more likely to produce significant academic growth, compared with simply encouraging students to elicit feedback from their teachers and peers (Mandouit and Hattie, 2023; Fleckenstein et al., 2024).

### 5.4 Gender difference of the mediating effect

The mediating effect of feedback self-efficacy between self-feedback behaviors and academic performance was further investigated on a gender basis. Multi-group structural equation modeling (MG-SEM) found significant differences among the specific predictive paths. This supplements empirical evidence on how male and female students could turn self-feedback behaviors into learning outcomes differently.

This finding is consistent with previous relevant studies, which report the gender differences in the effect between students’ self-assessment behaviors and academic outcomes (Bidjerano, 2005; Liu et al., 2021; Yang et al., 2023). This study found that male students with more frequent processing of feedback actions in their learning are more likely to be confident in their self-feedback capacities. In contrast, female students can more effectively translate their beliefs into self-feedback behaviors and academic proficiency. These findings might be sourced from the different cognitive and affective dynamics between male and female students. Previous studies report that male students often cognitively make evaluative judgements and reflect upon the obtained comments from their teachers and peers; their confidence in their knowledge and skills in employing self-feedback strategies in their learning process could be enhanced accordingly (Dumanjug et al., 2024). Female students are more likely to devote to the metacognitive and cognitive process when they perceive more confidence in their capacities for self-feedback, improving their learning outcomes (Guo, 2021; Ubago-Jimenez et al., 2024).

Interestingly, the predictive power of UF on feedback self-efficacy was almost the same across both gender groups, suggesting that feedback adoption is equally beneficial, regardless of gender. This finding reinforces the arguments that active use of feedback content remains a critical predictor of academic performance for both male and female students (Carless and Winstone, 2020).

This result supplements substantial empirical evidence of the critical role of gender differences in the self-feedback process (Huang, 2013; Lovász et al., 2022). Teachers should recognize that male and female students may engage in the self-feedback process differently, not only in cognitive processing but also in transforming feedback beliefs into academic outcomes (Matthews et al., 2009). More specialized feedback interventional strategies that support female students in developing their capacities for processing feedback and building male students’ feedback beliefs might improve the pedagogical effectiveness of feedback interventions and learning outcomes.

### 5.5 Research implications

This study provides several important implications of self-feedback, feedback self-efficacy, and academic proficiency for curriculum instructional practices within the context of Chinese high schools. First, the research finding that only UF, rather than SF or PF, could directly predict students’ academic proficiency underscores the idea that merely obtaining and interpreting feedback is insufficient in improving their learning. Instead, active use of feedback could supplement substantial learning attainments. This aligns with learner-centered feedback frameworks, emphasizing that feedback becomes influential only when students take proactive strategies to act upon it (Carless and Boud, 2018; Little et al., 2023).

Second, the significant mediating effects of feedback self-efficacy in the effect between self-feedback behaviors and academic proficiency support key assumptions from social cognitive theory (Bandura, 2011) and SRL models (Zimmerman, 2008). The findings indicate that students’ beliefs in their competencies of employing feedback effectively are imperative to translate their behavioral self-feedback engagement into learning achievement. This reinforces the need for teachers to teach students with feedback instructional strategies and improve their affective confidence with feedback.

Third, the gender-based analysis revealed nuanced differences in predictive pathways: male students showed a more substantial predictive effect between PF and feedback self-efficacy. In contrast, female students demonstrated a more substantial impact of feedback self-efficacy on academic proficiency. This suggests that gender may moderate the effectiveness of feedback-related strategies, highlighting the need for differentiated pedagogical approaches in different gender groups.

In summary, these findings advance our understanding of how self-feedback behaviors, mediated by feedback self-efficacy, could determine their academic outcomes. It also emphasizes the need to enhance students’ feedback beliefs, which could effectively translate the effects of the process and use feedback into academic outcomes (Winstone et al., 2021; Han, 2017). Additionally, the gender-differentiated instructional strategies shall be applied to maximize the effects of transforming self-feedback behaviors into learning achievements.

### 5.6 Limitations and future research

Despite the valuable findings in the present study, several limitations should also be recognized. First, this study employed a cross-sectional research design, which restricts the ability to capture the developmental trajectory and causal relationships among self-feedback behavior, feedback self-efficacy, and academic proficiency. As self-feedback behavioral processes are dynamic and evolve, longitudinal or experimental designs in future research would provide a more comprehensive understanding of how students’ feedback engagement influences academic outcomes across different stages of learning (Leenknecht and Carless, 2023; Li and Xue, 2023).

Second, the study relied primarily on self-reported questionnaires to measure all constructs, including self-feedback behaviors, feedback self-efficacy, and academic performance. Although self-report measures are widely used in SRL studies, they are subject to social desirability bias and introspective inaccuracy (Latkin et al., 2017; Teh et al., 2023). Future studies could incorporate multi-method approaches, such as classroom observations, teacher evaluations, performance-based assessments, or think-aloud protocols, to validate and enrich self-reported data.

Third, the sample was limited to high school students in a single city in China, which might constrain the generalizability of the research findings. Given the cultural emphasis in Chinese education on exam-driven performance and hierarchical classroom interactions (Liu and Feng, 2015; Guo and Xu, 2020), the effects of self-feedback, efficacy beliefs, and academic achievement may differ across other cultural or educational contexts. Future research should therefore apply this theoretical framework with participants from western cultural settings to examine the generality of the research findings across various cultural and geographical backgrounds.

## 6 Conclusion

The present study empirically investigates the predictive effects of self-feedback behaviors, feedback self-efficacy, and academic proficiency within a single structural framework in Chinese high schools. It further explores the gender differences among the said predictive effects. The findings showed that, among the three sub-actions of self-feedback, only UF could directly determine academic proficiency. This highlights the prominent role of taking proactive actions upon feedback in their learning process to achieve substantial academic growth. Furthermore, PF and UF could significantly impact students’ confidence in their feedback capacities and determine their learning performance. This empirically supports the critical role of feedback self-efficacy in mediating students’ self-feedback behaviors and learning achievements. Multi-group SEM further found that male students demonstrated a more substantial predictive effect of PF and feedback self-efficacy. In contrast, female students with stronger beliefs in feedback capacities were more likely to achieve substantial academic growth. These findings indicate that students’ gender might moderate self-feedback behaviors and learning performance. It provides evidence of teachers’ need for differentiated feedback strategies for male and female students.

The present study supplements robust empirical evidence to further our understanding of the complicated effect of self-feedback behavior, feedback self-efficacy, and learning achievements. It provides compelling evidence that teachers should support students with self-feedback, behavioral engagement, and improve their personal beliefs about their feedback. By doing this, students could better engage and benefit from the self-feedback process and achieve substantial learning attainment. This study also reveals that teachers should design differentiated feedback instructional strategies among male and female students, given the gender differences in the self-feedback effects.

## Data Availability

The raw data supporting the conclusions of this article will be made available by the authors, without undue reservation.

## Appendix A

### Feedback self-efficacy

1. I know the values of self-feedback.
2. I know how to implement self-feedback.
3. I can find materials to conduct self-feedback.
4. I know how to approach others for their comments.

### Seek feedback

1. I seek out examples of good work to improve my work.
2. I ask for comments about specific aspects of my work from others.
3. When I am working on a task, I consider comments I have received on similar tasks.
4. I seek feedback information from various learning resources.

### Process feedback

1. I carefully consider comments about my work before deciding whether to use them.
2. When receiving conflicting information from different sources, I judge what I will use.
3. When deciding what to do with comments, I consider the credibility of their sources.

### Use feedback

1. I can formulate my learning improvement plan after explicit inferences.
2. I would spend more time working on my weak areas.
3. I plan how I will use feedback to improve my future work, not just the immediate task.
4. I would continue seeking comments to improve my future learning.
